# Subdural Hematoma Presenting with Tinnitus After Spinal Anesthesia for Cesarean Section: A Case Report

**DOI:** 10.7759/cureus.84017

**Published:** 2025-05-13

**Authors:** Bülent M Çam, Semih Akar

**Affiliations:** 1 Department of Anesthesiology and Reanimation, Amasya University Sabuncuoglu Serefeddin Training and Research Hospital, Amasya, TUR; 2 Department of Neurosurgery, Amasya University Sabuncuoglu Serefeddin Training and Research Hospital, Amasya, TUR

**Keywords:** migraine, obstetric anesthesia, postdural puncture headache, spinal anesthesia, subdural hematoma, tinnitus

## Abstract

A 35-year-old woman with a history of migraine underwent cesarean section under spinal anesthesia using a 25G Quincke needle. On postoperative day two, she developed postdural puncture headache (PDPH) and bilateral tinnitus. Although the headache resolved with intravenous fluids and theophylline, tinnitus persisted. Audiometry revealed bilateral low-frequency sensorineural hearing loss, and cranial MRI confirmed bilateral subdural hematomas (SDHs). She was treated conservatively and discharged without neurological deficits. This case emphasizes the need to consider SDH in the differential diagnosis of PDPH, especially when atypical symptoms such as tinnitus are present. Early imaging is critical to avoid delayed diagnosis and unnecessary morbidity.

## Introduction

Spinal anesthesia is a commonly preferred and effective regional anesthesia technique in obstetric surgery. One of its common but typically benign complications is postdural puncture headache (PDPH), which occurs in approximately 10% to 40% of patients undergoing lumbar puncture. However, the incidence may be as low as 2% when small-gauge (≤24 gauge), pencil-point spinal needles are used [1]. According to the International Headache Society (IHS), PDPH is defined as a headache developing within five days after a lumbar puncture and attributed to cerebrospinal fluid (CSF) leakage through the dural breach [2]. The underlying pathophysiology of PDPH is primarily linked to CSF leakage at the puncture site, which may exceed the rate of normal CSF production. This loss results in intracranial hypotension, causing traction and downward displacement of pain-sensitive intracranial structures, particularly the meninges. As a result, patients experience orthostatic headache along with potential neurological manifestations due to cranial or upper cervical nerve involvement, such as nausea, dizziness, neck stiffness, visual disturbances, and, less commonly, tinnitus, hearing loss, or radicular pain in the upper limbs [1]. Although the positional component is not a strict diagnostic criterion, PDPH is typically characterized by bilateral frontal or occipital headache that worsens when upright and improves in the supine position [2].

Despite PDPH’s typically benign course, rare yet serious complications like subdural hematoma (SDH) may occur [3, 4]. In a study including 56 case reports of SDH following spinal anesthesia, PDPH was identified in 60% of the cases prior to the development of subdural bleeding [5]. SDH is a potentially life-threatening condition, particularly when its symptoms mimic or overlap with those of PDPH, potentially delaying diagnosis. In the literature review presented by Amorim et al., which evaluated 35 cases of SDH following neuraxial block, surgical intervention was required in 77% of the patients (27 cases), and four patients died [6]. The term neuraxial block broadly encompasses both spinal and epidural anesthesia techniques. In spinal anesthesia, local anesthetics are injected into the subarachnoid space, beyond the dural membrane. In contrast, epidural anesthesia involves the administration of medication into the epidural space, located just outside the dura mater. The most widely accepted mechanism for the development of SDH is intracranial hypotension, as in PDPH [5]. This condition leads to downward displacement of brain structures, causing stretching and tearing of the bridging veins, which in turn results in bleeding into the subdural space. Yamashima and Friede, through electron microscopic analysis, identified key structural vulnerabilities in the bridging veins of the subdural space-namely, the presence of exceptionally thin walls, disorganized collagen fibers, and the absence of external arachnoid trabecular support. They further noted that venous terminations firmly affixed to the dura mater exhibit greater positional stability than those connected to the more dynamic brain parenchyma. This anatomical arrangement predisposes the subdural vein segments to traction-induced rupture during shifts in head position or intracranial pressure dynamics [7].

Although the underlying mechanisms are similar, the development of SDH following neuraxial block is exceedingly rare. In a retrospective survey conducted in 1990, subdural hemorrhage was reported in only one out of 506,000 pregnant patients who underwent epidural block [8]. More recently, a 2014 case series demonstrated that the incidence of SDH following epidural anesthesia can rise to as high as 1.1% in the presence of an unintentional dural puncture [9]. It is important to note that this rate is based on cases where the dura was punctured by an epidural needle. Even so, this is a rather serious rate and clearly highlights the need for greater attention to this complication. The exact incidence of SDH following spinal anesthesia, however, remains unknown.

In this report, we present a case of intracranial SDH that developed following spinal anesthesia, with an initial clinical presentation of PDPH accompanied by persistent tinnitus.

## Case presentation

Written informed consent was obtained from the patient for this report. The CAse REport (CARE) guideline was followed in the preparation and reporting of this case.

A 35-year-old woman of Turkish ethnicity, gravida 2 para 1, at 39 weeks of gestation, with a height of 158 cm and a weight of 65 kg, was scheduled for emergency cesarean section due to meconium-stained amniotic fluid. She was a homemaker. Her medical history was notable for migraine, for which she reported using paracetamol during pregnancy. She had no known psychosocial or genetic disorders. There was no history of pregnancy-induced hypertension (PIH), gestational diabetes mellitus (GDM), or other perinatal complications. Preoperative vital signs were stable: temperature of 36.8°C, blood pressure of 114/72 mmHg, heart rate of 88 beats per minute, respiratory rate of 14 breaths per minute, and oxygen saturation (SpO₂) of 99%. International normalized ratio (INR) and other coagulation parameters were within normal limits. The patient had no history of any anticoagulant use.

Spinal anesthesia was applied with a midline technique from the L3-L4 intervertebral space with the patient in a sitting position with a 25G Quincke needle, and access to the subarachnoid space was achieved in a single attempt. During the procedure, the CSF flow was clear, and no complications were observed. Anesthesia was provided with 2.4 ml (12 mg) of 0.5% hyperbaric bupivacaine. No complications were observed during the operation.

On postoperative day 2, the patient developed a diffuse, non-localized, moderate-intensity headache accompanied by bilateral tinnitus. Given that the headache worsened in the upright position and improved when lying down, PDPH was considered the preliminary diagnosis. However, it is important to consider migraine as a differential diagnosis in postpartum patients presenting with headache and tinnitus. According to the International Classification of Headache Disorders, migraine is defined as a recurrent headache disorder with attacks lasting four to 72 hours, typically unilateral and pulsating, of moderate-to-severe intensity, and aggravated by routine physical activity [2]. It may be accompanied by nausea, photophobia, and phonophobia. Notably, some migraine subtypes, such as vestibular migraine or migraine with brainstem aura, can present with auditory symptoms, including tinnitus and sensorineural hearing loss [10]. Moreover, migraine symptoms often improve during pregnancy but may recur in the early postpartum period due to hormonal shifts [11]. This overlap in clinical features can obscure the recognition of more serious complications such as subdural hematoma. In our case, although the patient had a history of migraine, the fact that the headache was pulseless and position-dependent excluded the diagnosis of migraine.

According to the Consensus Practice Guidelines on Postdural Puncture Headache from a multisociety, international working group, the initial approach to PDPH should be conservative. Intravenous fluids and caffeine therapy are recommended as first-line treatments [12]. However, in cases where caffeine is not available, both theophylline and aminophylline have also been shown to be effective alternatives [13]. In our case, conservative treatment consisted of administering 2000 mL of intravenous saline over two hours and 200 mg of intravenous theophylline over 30 minutes. On neurological examination, the patient was alert, oriented, and cooperative. Cranial nerve examination was unremarkable. The pupils were equal and reactive to light. Motor strength and sensation were preserved in all extremities. No focal deficits were detected. There was no nuchal rigidity.

While the headache improved with treatment, the persistence of tinnitus prompted us to consider alternative differential diagnoses. Although postpartum preeclampsia was initially considered due to the bilateral nature of the headache and a normal neurological examination, this diagnosis was ruled out based on the absence of systemic hypertension and lack of proteinuria in the urine.

**Figure 1:**
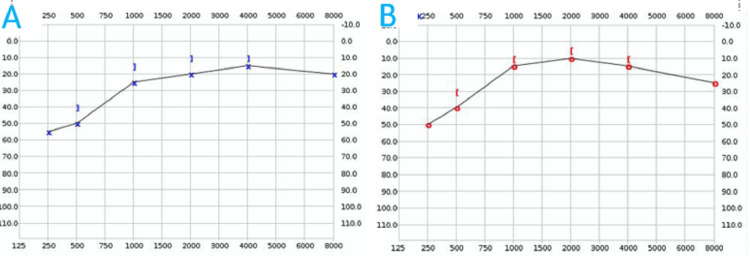
Audiometric test results A) Left ear; B) Right ear

Since tinnitus persisted, audiometric evaluation was performed on the third postoperative day. Pure-tone audiometry of the left ear demonstrated low-frequency sensorineural hearing loss. The hearing threshold was 55 dB HL at 250 Hz and 45 dB HL at 500 Hz. Improvement was observed at mid and high frequencies, with thresholds around 30 dB HL at 1000 Hz, 25 dB HL at 2000 Hz, 20 dB HL at 4000 Hz, and 25 dB HL at 8000 Hz (Figure 1A). The right ear also exhibited low-frequency sensorineural hearing loss, with thresholds of 50 dB HL at 250 Hz and 40 dB HL at 500 Hz. Similar to the left ear, hearing improved at higher frequencies: 25 dB HL at 1000 Hz, 20 dB HL at 2000 Hz, 25 dB HL at 4000 Hz, and 30 dB HL at 8000 Hz at 250, 500, and 1000 Hz (Figure 1B). The bilateral nature of the hearing loss made the diagnosis of Ménière’s disease less likely. Upon the recommendation of the relevant clinic physician, systemic methylprednisolone therapy was initiated.

**Figure 2:**
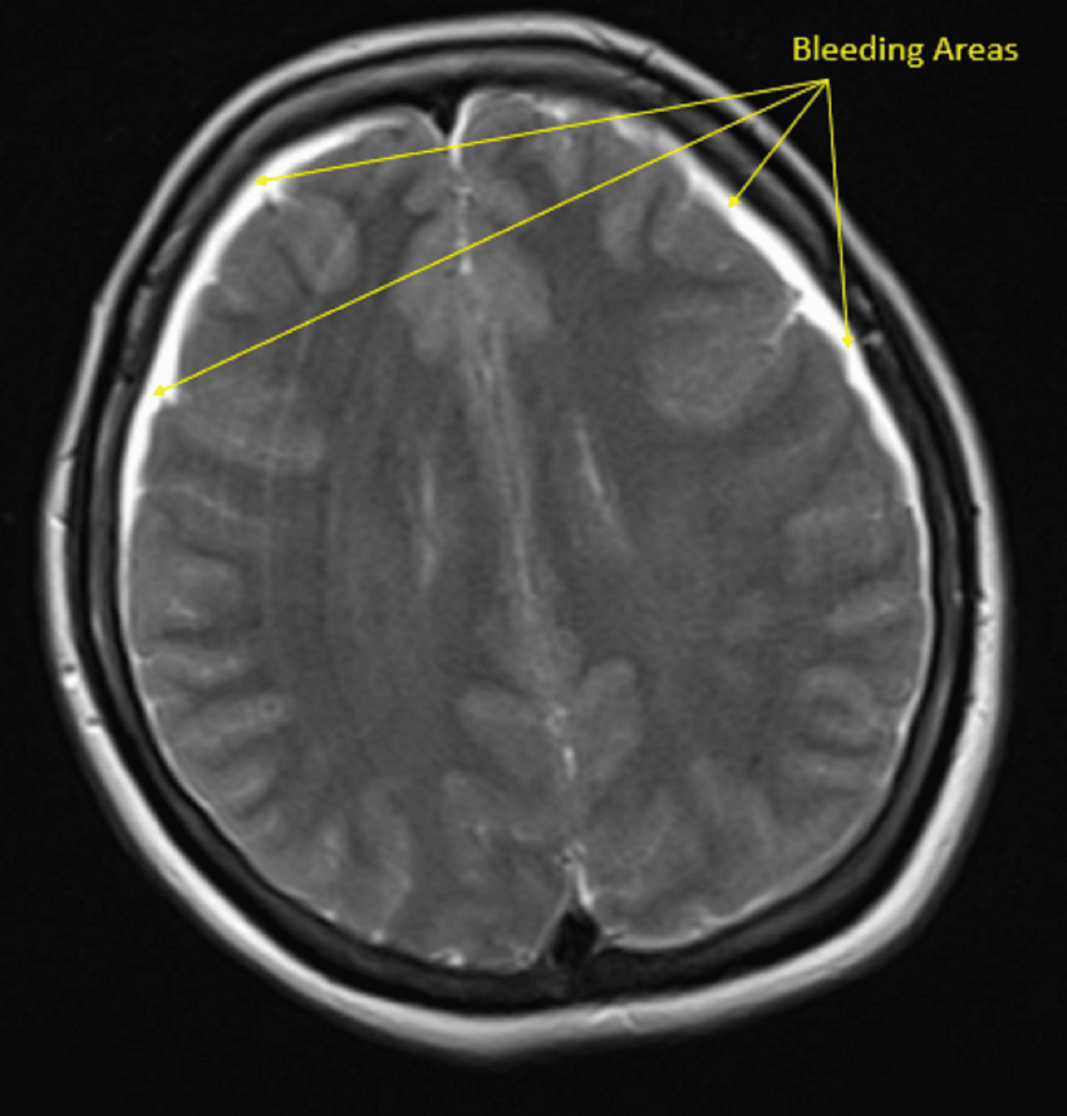
Bilateral subdural hematoma observed in the magnetic resonance image (MRI) obtained on the fifth postoperative day The arrows indicate the bleeding areas.

**Figure 3:**
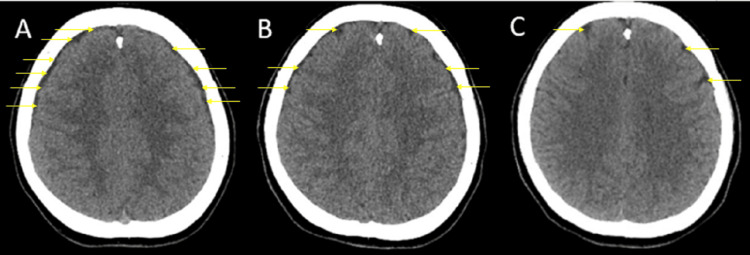
Computed tomography images A) Bilateral subdural hematoma on the fifth postoperative day; B) Bilateral subdural hematoma had regressed slightly on the sixth postoperative day; C) Almost completely resolved subdural hematoma on the eighth postoperative day The arrows indicate the bleeding areas. Serial imaging demonstrates a progressive reduction in the subdural hematoma.

Due to the persistence of tinnitus, cranial imaging was performed on postoperative day 5. Cranial computed tomography (CT) and magnetic resonance imaging (MRI) revealed bilateral SDHs appearing as thin, film-like collections approximately 3-4 mm in thickness over the frontoparietal regions of both cerebral hemispheres (Figures 2- 3A). These findings were consistent with SDH. There was no evidence of midline shift, mass effect, cerebral edema, or infarction.

Generally, surgical evacuation is indicated when the hematoma thickness exceeds 10 mm or when there is a midline shift greater than 5 mm, regardless of the Glasgow Coma Scale (GCS) score. Additionally, surgery is recommended in patients with a GCS score below nine, asymmetric or fixed dilated pupils, or intracranial pressure exceeding 20 mmHg [14]. As none of these criteria were met in our patient, a conservative management approach was deemed appropriate. By the evening of postoperative day 5, the patient reported complete resolution of tinnitus.

During conservative follow-up, serial CT scans demonstrated marked regression of the bilateral subdural collections by postoperative day 6. On postoperative day 8, the CT images showed that the subdural collection in the right parietotemporal region had nearly completely resolved (Figure 3). This radiological improvement paralleled the patient’s clinical course and further supported the effectiveness of the conservative treatment strategy.

**Figure 4:**
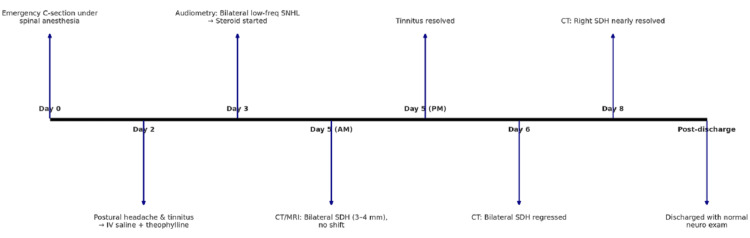
Infographic timeline of clinical events SDH: subdural hematoma

Daily neurological assessments revealed that the patient remained fully alert and oriented, with intact cranial nerve function and preserved motor and sensory findings. No new neurological deficits were identified. Following confirmation of both clinical and radiological improvement, the patient was discharged and scheduled for outpatient follow-up. The timeline is shown in Figure 4.

## Discussion

SDH is an exceedingly rare yet potentially life-threatening complication following neuraxial anesthesia. While the mechanisms underpinning its development, chiefly intracranial hypotension secondary to CSF leakage, are well described, its diagnosis remains challenging due to clinical overlap with PDPH symptoms.

In our case, the presence of bilateral tinnitus with a non-pulsatile, positional headache in the early postoperative period raised suspicion, particularly after the headache resolved while the auditory symptom persisted. While previous reports have highlighted SDHs presenting with nausea, visual disturbances, altered mental status, or hemiparesis, auditory symptoms like tinnitus have been rarely emphasized, and our case contributes to this gap [4,15-17].

Moreover, although PDPH is typically self-limiting, the persistence of atypical symptoms such as sensorineural hearing loss prompted early imaging, which revealed bilateral SDH. This finding supports the assertion by Joudi and Ansari that a change in the characteristic pattern of PDPH, particularly with the appearance of non-classical symptoms, should prompt timely imaging [11].

Another notable aspect of our case is the coexistence of a prior migraine diagnosis. While migraine-associated auditory symptoms are well documented, our patient's non-pulsatile, posture-sensitive pain and audiometric confirmation of low-frequency bilateral sensorineural hearing loss deviated from typical migraine presentations. This distinction underscores the importance of not anchoring to preexisting diagnoses when evaluating postoperative complications.

Patients with PDPH typically describe a headache that worsens when sitting or standing and improves while lying down. However, according to the IHS, the positional component is no longer considered a diagnostic criterion. Therefore, any headache occurring after neuraxial anesthesia should prompt careful evaluation for PDPH and other potentially serious complications. The presence of neurological symptoms such as tinnitus, diplopia, nausea, or mental status changes necessitates a thorough differential diagnosis. It should also be noted that SDH may occur even in the absence of PDPH. Nevertheless, this remains a rare occurrence. SDH has been reported to occur 19 times more frequently in cases accompanied by PDPH than in those without it [11].

In a 2021 study by Joodi and Ansari investigating PDPH in pregnant patients, the authors proposed a list of red flags indicating the need for advanced imaging. Notably, tinnitus and muffled hearing were excluded from the neurological deficits listed, whereas a change in the characteristic pattern of PDPH was included as a warning sign [11]. When PDPH following spinal anesthesia changes in character over time, advanced imaging modalities such as CT and MRI should not be delayed.

Although most cases of SDH have been reported in association with obstetric surgeries, there are also reports linked to various other types of surgical procedures [15-17]. It has even been documented in a six-week-old infant following spinal anesthesia [18]. However, physiological alterations in the postpartum period may increase the susceptibility of obstetric patients to such complications. Cases of SDH occurring concurrently with pituitary apoplexy have also been reported, and it is suggested that changes in intracranial pressure may affect pituitary perfusion [19]. Additionally, a case resulting in brain death has been described in the literature [20].

A key strength of this case lies in the clinical vigilance that led to the early identification of SDH despite the confounding presence of a prior migraine diagnosis. In our case, full recovery was achieved through conservative management without the need for surgical intervention. The nature of the headache and the early onset of tinnitus acted as clinical warning signs, prompting the timely use of further neuroimaging techniques.

## Conclusions

This case highlights the importance of considering SDH in patients with postdural puncture headache after spinal anesthesia. Although symptoms such as tinnitus can also occur in migraine, their persistence may warrant careful evaluation rather than immediate attribution to a preexisting condition. In selected cases, timely imaging might aid in avoiding delayed diagnosis of rare but serious complications. In our patient, early diagnosis, a multidisciplinary approach, and conservative management resulted in full recovery without any neurological sequelae.
